# Internalizing and externalizing problems in childhood and adolescence as predictors of work incapacity in young adulthood

**DOI:** 10.1007/s00127-017-1409-6

**Published:** 2017-06-21

**Authors:** Jurgita Narusyte, Annina Ropponen, Kristina Alexanderson, Pia Svedberg

**Affiliations:** 1 Division of Insurance Medicine, Department of Clinical Neuroscience, Karolinska Institute, 17177 Stockholm, Sweden; 2 Finnish Institute of Occupational Health, Helsinki, Finland

**Keywords:** Sick leave, Internalizing and externalizing problems, Childhood, Adolescence, Disability pension

## Abstract

**Background:**

There is limited information regarding the association between youth mental health problems and work incapacity in adulthood. We investigated whether internalizing (depressive, anxious, somatic complaints) and externalizing (aggressive, rule-breaking) behavior problems in childhood and adolescence were associated with sickness absence (SA) and disability pension (DP) in young adulthood.

**Methods:**

Data were used from the population-based and prospective Twin Study of Child and Adolescent Development (TCHAD) which includes all Swedish twins born in 1985–1986 (*N* = 2570). Internalizing and externalizing behavior problems were assessed with the Child Behavior Checklist at ages of 8–9, 13–14, 16–17, and 19–20 years. Individuals participating in TCHAD were followed regarding SA and DP during 2001–2013 using nationwide registers. Cox regression models were applied to assess hazard ratios (HR) with 95% confidence intervals (CI).

**Results:**

Each one-unit increase of rule-breaking behavior implied a significant higher risk for SA in early adulthood, despite of age at assessment, with the highest HR of 1.12 (95% CI 1.05–1.19) at age of 8–9 years. Higher levels of anxious and depressive symptoms in childhood and adolescence were associated with DP in early adulthood despite age at assessment, with the highest risk at age 19–20 years [HR 1.31 (95% CI 1.12–1.53)]. The associations attenuated slightly when familial factors were taken into account.

**Conclusions:**

Internalizing and externalizing behavior problems identified at an early age (8–9 years) increased risk for SA and DP in young adulthood. These findings indicate that early prevention and intervention efforts to reduce behavior problems may promote a successful start in working life.

## Introduction

Marginalization from work life due to work incapacity is a public health concern in several European countries [1]. In Sweden, work incapacity in terms of sickness absence (SA) and disability pension (DP), has been increasing among young adults (i.e., <29 years of age) during the last decade [2]. As one of the ways to tackle this trend, OECD has recommended early intervention and support of school-age children experiencing mental health problems [3]. However, knowledge is lacking on what type of mental health problems that post the highest risk for future work incapacity, and is needed to ensure a smoother transition into the labor market.

Few studies have investigated the association between early-life factors, including mental health problems in childhood and adolescence, and work disability in adulthood. For example, self-reported physical and mental health problems in adolescence have been shown to be associated with receiving social and medical benefits due to work disability in young adulthood [4–8]. In these studies, the main focus has been on depression and anxiety, whereas a broader range of mental health problems, including behavioral and emotional problems, has not yet been studied. Also, work incapacity was measured as receiving social and medical benefits, which included both benefits due to work disability and unemployment. However, possible differences between SA and DP were not studied. In sum, knowledge is lacking on whether different behavioral and emotional problems in childhood and adolescence may have different impact on future work disability as well as whether the associations may depend on the age when these problems were experienced.

The continuity of mental health problems, including behavioral and emotional problems, in childhood and/or adolescence into adulthood is well acknowledged (e.g., [9]). Both internalizing (e.g., depression, anxiety, somatic complaints) and externalizing (e.g., aggression, delinquency) behavior problems have been found to persist into adulthood, although different predictive paths have been observed. For example, internalizing behavior problems were suggested to have homotypic continuity (i.e., predicts the same disorder over time), whereas externalizing behavior problems demonstrated heterotypic (i.e., predicts another disorder over time) prediction of psychopathology in adulthood [10–12]. In terms of work disability, little is still known on whether internalizing and externalizing behavior problems may be differently associated to SA and/or DP in early adulthood.

A twin study design is a powerful tool to account for unmeasured familial (i.e., genetic and shared environmental) influences on an association. Twins in a pair are optimally matched on genetic and shared environmental factors as well as on their age and sex (for the same-sexed pairs). Adjusting for familial influences diminishes a possibility for erroneous conclusions concerning effects from risk factors of interest. For example, the association between behavior problems and SA/DP may arise due to common genetic and shared environmental susceptibility between behavior problems and SA/DP. Alternatively, behavior problems could have a direct effect for development of SA/DP. Genetic factors explain a moderate portion of the variance (40–50%) in SA and DP (e.g., [13–15]). Previous studies have also found moderate to high genetic influences on internalizing as well as on externalizing behavior problems (e.g., [16, 17]). Further, one study reported that the associations between personality disorders and work disability were explained by genetic and non-shared environmental factors [18] in young adults. In sum, the association between internalizing and externalizing behavior problems in adolescence and work incapacity in adulthood may be confounded by genetic and/or shared environmental factors and more knowledge is warranted on this.

The aim of the present study was to examine whether occurrence of internalizing and externalizing behavior problems at different ages across childhood and adolescence increased risk for SA and DP in young adulthood, also adjusting for familial confounding.

## Methods

### Sample

A population-based prospective cohort study including all twins from the Swedish Twin Registry who were born in Sweden 1985–1986 (*N* = 2960) [19] was conducted. These twins were invited to participate in a longitudinal Twin study of CHild and Adolescent Development (TCHAD), see [20]. Twins and/or their parents were contacted on four occasions (i.e., Waves); when the twins were 8–9, 13–14, 16–17, and 19–20 years old. The participants were mailed a questionnaire including extensive batteries of questions on physical health as well as emotional and behavior problems. The response rates were 75% (*n* = 1339 for parents reporting information on their twins), 78% (*n* = 2263 for twin self-reports), 82% (*n* = 2369 twins), and 59% (*n* = 1698 twins) at Waves 1–4, respectively.

In Sweden, all residents who have income from work or unemployment benefits are from the age of 16 years entitled to sickness benefits from the Swedish National Social Insurance Agency, if unable to work due to disease or injury. DP can be granted to those aged 16 (and from 2003 to those aged 19), who, due to disease or injury, have long-term or permanently reduced work capacity, even if not having had any previous income. Since 2003, DP can also be granted to young adults for prolonged schooling in order to finish elementary or secondary school. For all twins, data on date of SA and DP were obtained from the Swedish National Social Insurance Agency for the years 2001–2013. For those working, the employer in most cases provides sick pay for the first 14 days of a SA spell, why the register data from the Agency do not include information about SA spells ≤14 days. Data on diagnoses for SA were available for years 2005–2013 and for DP 2001–2013. All register data were linked to the twins, using the unique ten-digit personal identification number assigned to all residents in Sweden.

After excluding individuals with missing questionnaire data at all waves or with missing information on zygosity (*n* = 246), or who were later granted DP due to mental retardation diagnoses (ICD 10: F70–F79, *n* = 18), or who died before they turned 16 years (*n* = 2); the final sample comprised of 2570 twins, whereof 1029 were monozygotic and 1541 dizygotic twins.

### Exposure variables

The presence of internalizing and externalizing behavior problems was assessed through the Child Behavior Checklist (CBCL) [21]. The CBCL is a reliable and valid instrument for assessment of behavioral and emotional problems in children and adolescents [21]. The CBCL includes 102 items with a three-point Likert-scale response-format that can be summarized into three scales: Internalizing, Externalizing, and Total problems scales. Internalizing and Externalizing scales include syndrome scales, or subscales, referring to the sets of co-occurring problems [21]. In the internalizing scale, the syndromes are grouped as Depressed/Anxiety (score range 0–28), Withdrawal/Depressed (score range 0–14), and Somatic complaints (score range 0–18). The externalizing scale includes Aggressive Behavior (score range 0–38) and Rule-Breaking (Delinquent) Behavior (score range 0–22) syndromes. The CBCL was administered to the twins’ parents in Wave 1 and an adapted self-report version of CBCL, Youth-Self Report [21], was mailed to twins in Waves 2–4.

### Outcome variables

The following two outcomes were used: having a SA spell (yes/no) or being granted DP (yes/no) during follow-up though 2013.

In Sweden, all residents aged 16–65 years who have income from work or unemployment benefits are entitled to sickness benefits from the Social Insurance Agency, if unable to work due to disease or injury. Among employed individuals, sick pay is in most cases paid by the employer during the first 14 days of a sick-leave spell, which means that we do not have data on most of the short sick-leave spells. DP can be granted to those who, due to disease or injury, have permanently reduced work capacity, even if not having previous income from work and may be granted for both full- and part-time absence.

### Follow-up time

**Fig. 1:**
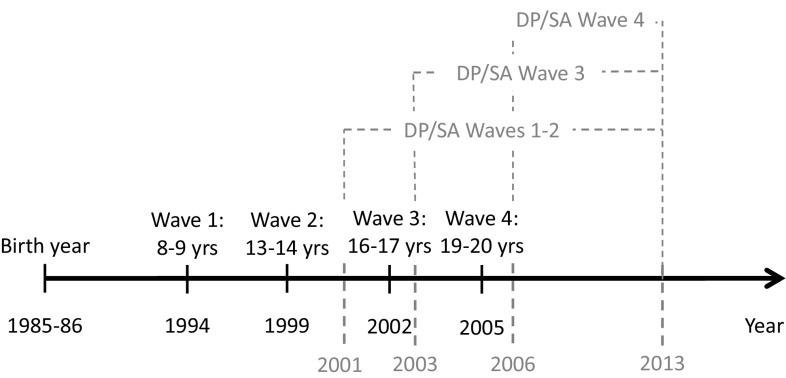
The follow-up times of the cohort

All individuals were followed until the date of the first SA-spell or the date of granted DP, respectively, until emigration, death, or the last date of follow-up, 2013-12-31. In the analyses of associations with behavior problems at Wave 1 and 2, the individuals were followed regarding SA or DP, respectively, from the year they turned 16 years (i.e., year 2001 or 2002 depending on the birth year of the participants). For analyses of behavior problems at Waves 3 and 4, the follow-up began the year after the assessment of behavior problems. That is, for the analyses of behavior problems measured at Wave 3, the follow-up period began in 2003, whereas for analyses of behavior problems measured at Wave 4, the follow-up began in 2006. Individuals that were granted DP before the measurements of behavior problems at Wave 3 (*n* = 8) or 4 were excluded from the data analyses (*n* = 29). The follow-up times depending on the wave of assessment are outlined in Fig. 1.

### Statistical analyses

Descriptive statistics of the sample were computed including frequencies, percentages, and means with standard deviations. Cox proportional hazard regression models were applied to estimate the association between the subscales of internalizing and externalizing behavior problems at different ages and occurrence of SA and DP in adulthood. The analyses were clustered on twin pair identity to adjust for twin dependency within pairs. The subscales were treated as continuous variables and hazard ratios (HR) were calculated of being on SA or DP during the follow-up. The analyses were also adjusted for sex and parental education (measured by a question to both parents: “What is mother’s/father’s education?”). Influence of familial factors was tested by running conditional Cox proportional hazards analyses that only included twin pairs discordant for SA or DP, respectively. Twin pairs were treated as discordant if one twin in a pair was granted SA or DP during the follow-up period, respectively, whereas the co-twin was not. In the analyses of SA, those discordant twin pairs where one twin was on SA and the other one on DP, were removed from the analyses. Familial factors are suggested to influence the association if HRs computed in conditional analyses differ from HRs computed in the whole sample [22].

All statistical analyses were performed with SAS 9.4 [23].

## Results

Mean levels for internalizing behavior problems were consistently higher among girls than boys, whereas mean levels for externalizing behavior problems were slightly higher among boys, Table 1. Levels of behavior problems were higher among those participants who were on SA or DP during follow-up as compared to levels of behavior problems among all participants (Table 1).

**Table 1:** Number of responders and means (standard deviations) for Child Behavior Check List (CBCL)-subscales at Waves 1–4 among all study participants and those who were on sickness absence (SA) or granted disability pension (DP) during follow-up 2001–2013

Subscale	*n*	Wave 1	*n*	Wave 2	*n*	Wave 3	*n*	Wave 4
*Women*								
All								
Anxiety/depressed	1038	1.97 (2.89)	1104	5.14 (4.62)	1193	5.67 (5.00)	978	8.37 (6.73)
Withdrawn/depressed	1040	1.05 (1.30)	1115	2.51 (1.96)	1194	2.82 (2.16)	980	1.80 (2.33)
Somatic complaints	1042	0.69 (1.22)	1096	2.23 (2.39)	1184	2.37 (2.52)	987	3.32 (3.36)
Rule-breaking behavior	1037	0.75 (1.28)	1107	2.50 (2.20)	1191	2.93 (2.33)	978	1.80 (2.17)
Aggressive behavior	1038	4.59 (4.81)	1103	7.99 (4.39)	1190	7.74 (4.36)	978	5.08 (3.85)
SA 2001–2013								
Anxiety/depressed	272	2.01 (2.84)	292	5.27 (4.68)	317	6.07 (5.34)	259	9.06 (6.82)
Withdrawn/depressed	273	1.05 (1.25)	294	2.60 (2.05)	316	2.85 (2.16)	260	2.00 (2.49)
Somatic complaints	273	0.74 (1.25)	295	2.59 (2.62)	315	2.83 (2.68)	264	3.95 (3.57)
Rule-breaking behavior	272	0.91 (1.37)	292	2.65 (2.19)	316	3.09 (2.30)	259	1.84 (1.95)
Aggressive behavior	272	5.28 (5.18)	292	8.20 (4.56)	315	7.93 (4.21)	259	5.33 (3.92)
DP 2001–2013								
Anxiety/depressed	32	2.56 (3.29)	31	7.45 (6.32)	31	7.65 (6.96)	25	15.48 (9.69)
Withdrawn/depressed	32	1.56 (2.06)	32	3.34 (2.50)	32	3.41 (2.95)	26	3.92 (3.47)
Somatic complaints	32	1.19 (1.99)	30	2.63 (3.33)	31	3.13 (3.43)	26	6.73 (4.68)
Rule-breaking behavior	32	1.06 (2.06)	31	3.10 (2.47)	31	3.39 (2.69)	25	3.52 (4.01)
Aggressive behavior	32	5.19 (5.13)	30	8.47 (5.04)	31	7.74 (5.10)	25	6.60 (4.66)
*Men*								
All								
Anxiety/depressed	1048	1.62 (2.48)	1051	3.57 (3.55)	1057	2.86 (3.31)	633	4.59 (5.05)
Withdrawn/depressed	1050	0.94 (1.21)	1057	2.01 (1.81)	1065	1.99 (1.87)	633	1.55 (2.02)
Somatic complaints	1053	0.54 (1.02)	1047	1.42 (1.73)	1054	1.22 (1.67)	637	1.44 (2.04)
Rule-breaking behavior	1048	1.09 (1.43)	1046	2.80 (2.18)	1055	3.00 (2.28)	633	2.12 (2.43)
Aggressive behavior	1048	5.27 (5.11)	1050	7.89 (4.82)	1058	7.10 (4.49)	633	3.58 (2.84)
SA 2001–2013								
Anxiety/depressed	196	1.60 (2.65)	200	3.78 (3.70)	195	2.87 (3.19)	106	4.81 (5.17)
Withdrawn/depressed	197	0.99 (1.37)	200	2.05 (1.94)	197	1.97 (1.71)	106	1.52 (2.04)
Somatic complaints	197	0.63 (1.21)	201	1.44 (1.77)	192	1.20 (1.63)	107	1.90 (2.44)
Rule-breaking behavior	196	1.41 (1.81)	198	3.23 (2.54)	194	3.61 (2.18)	106	3.03 (3.14)
Aggressive behavior	196	6.37 (6.01)	200	8.53 (5.39)	196	7.83 (5.01)	106	4.19 (3.62)
DP 2001–2013								
Anxiety/depressed	23	3.04 (2.79)	22	5.23 (4.55)	19	4.89 (4.97)	7	10.86 (7.63)
Withdrawn/depressed	23	1.78 (1.78)	22	2.86 (2.23)	20	3.40 (2.80)	7	3.29 (2.14)
Somatic complaints	23	0.48 (0.90)	22	1.73 (2.29)	19	1.26 (1.33)	7	1.86 (2.91)
Rule-breaking behavior	23	0.87 (1.74)	22	2.59 (1.76)	18	2.56 (1.89)	7	1.86 (2.27)
Aggressive behavior	23	4.87 (4.86)	22	8.23 (5.01)	19	6.16 (4.80)	7	5.14 (2.85)

Cumulative incidences of SA and DP during the different follow-up periods are presented in Table 2. Approximately 27% of the women and 18% of the men had at least one SA spell. In both women and men, DP was granted to 3, 3 and 2% of the individuals during the follow-up periods 2001–2013, 2003–2013, and 2006–2016, respectively.

**Table 2:** Cumulative incidence of sickness absence and disability pension among women and men during the years 2001–2013, 2003–2013, and 2006–2013

	Follow-up, years					
	Women			Men		
	2001–2013 (*n* = 1321)	2003–2013 (*n* = 1315)	2006–2013 (*n* = 1292)	2001–2013 (*n* = 1249)	2003–2013 (*n* = 1245)	2006–2013 (*n* = 1222)
Sickness absence						
Number of cases (%)	357 (27.0)	356 (27.1)	345 (26.7)	236 (18.9)	236 (19.0)	223 (18.3)
Mean follow-up, years (SD)	11 (2.1)	10 (2.0)	8 (1.6)	11 (2.1)	10 (2.0)	8 (1.7)
Disability pension						
Number of cases (%)	40 (2.9)	36 (2.7)	20 (1.5)	35 (2.7)	31 (2.4)	17 (1.3)
Mean follow-up, years (SD)	12 (1.7)	11 (1.4)	8 (1.0)	12 (1.5)	11 (1.2)	8 (0.9)

Few significant associations were observed between internalizing behavior problems and SA, whereas for externalizing behavior, specifically Rule-breaking behavior was associated with future SA at all waves of assessment (Table 3). Significant associations were found between each one-unit increase in Anxiety/Depressed and SA for Waves 3 and 4 as well as between Somatic complaints and SA at Waves 2 and 4. The associations attenuated when adjusting for parental education and sex except for Somatic complaints at wave 4. Rule-breaking at Waves 1–4 was significantly associated to SA in adulthood, also after adjusting for sex and parental education. The HRs varied between 1.06 and 1.12 which implies an increase in risk with each one unit increase in the subscale. Aggressive Behavior measured at Wave 1 and 4 was associated to SA. After adjusting for sex and parental education, only the association at Wave 1 remained significant. In the analyses of discordant twins, HRs changed slightly.

**Table 3:** Crude and adjusted each one-unit increase hazard ratios (HR) and 95% confidence intervals (CI) for a new sickness absence spell in 2001–2013 following Internalizing and Externalizing behavior problems during childhood and adolescence

Scale	Crude model for all individuals		Adjusted model for all individuals		Conditional model for discordant twin pairs	
	*n*	HR (95% CI)	*n*	HR^a^ (95% CI)	*n*	HR^b^ (95% CI)
*Internalizing problems*						
Anxiety/depressed						
Wave 1	2079	1.01 (0.98–1.04)	1790	1.00 (0.96–1.04)	646	1.02 (0.95–1.11)
Wave 2	2147	1.02 (1.00–1.04)	1872	1.01 (0.99–1.03)	670	1.01 (0.96–1.06)
Wave 3	2239	**1.03 (1.01**–**1.05)**	1986	1.01 (0.99–1.03)	694	1.03 (0.99–1.08)
Wave 4	1586	**1.03 (1.01**–**1.04)**	1390	1.01 (0.99–1.03)	460	1.02 (0.98–1.07)
Withdrawn/depressed						
Wave 1	2083	1.04 (0.96–1.11)	1794	1.02 (0.94–1.10)	648	0.98 (0.84–1.14)
Wave 2	2164	1.04 (0.99–1.08)	1885	1.02 (0.97–1.07)	675	0.97 (0.88–1.07)
Wave 3	2247	1.02 (0.98–1.07)	1993	0.99 (0.94–1.04)	698	1.02 (0.94–1.12)
Wave 4	1588	1.04 (1.00–1.09)	1392	1.02 (0.97–1.07)	460	1.04 (0.93–1.17)
Somatic complaints						
Wave 1	2088	**1.08 (1.01**–**1.17)**	1799	1.06 (0.98–1.16)	649	1.04 (0.88–1.23)
Wave 2	2135	**1.07 (1.03**–**1.11)**	1859	**1.05 (1.01**–**1.10)**	673	1.03 (0.95–1.12)
Wave 3	2227	**1.08 (1.04**–**1.12)**	1977	**1.05 (1.01**–**1.09)**	693	1.04 (0.96–1.12)
Wave 4	1599	**1.09 (1.06**–**1.12)**	1402	**1.06 (1.02**–**1.09)**	463	**1.11 (1.02**–**1.22)**
*Externalizing problems*						
Rule-breaking behavior						
Wave 1	2078	**1.12 (1.06**–**1.19)**	1789	**1.12 (1.05**–**1.19)**	645	1.06 (0.90–1.25)
Wave 2	2145	**1.06 (1.02**–**1.10)**	1865	**1.04 (1.00**–**1.09)**	669	1.02 (0.93–1.11)
Wave 3	2236	**1.07 (1.03**–**1.10)**	1983	**1.06 (1.02**–**1.10)**	695	1.01 (0.93–1.09)
Wave 4	1586	**1.05 (1.01**–**1.10)**	1390	**1.05 (1.00**–**1.10)**	460	1.03 (0.93–1.14)
Aggressive behavior						
Wave 1	2079	**1.04 (1.02**–**1.05)**	1790	**1.03 (1.01**–**1.05)**	646	1.03 (0.98–1.08)
Wave 2	2145	**1.02 (1.00**–**1.04)**	1869	1.01 (0.99–1.03)	673	0.98 (0.94–1.02)
Wave 3	2237	**1.02 (1.00**–**1.04)**	1985	1.01 (0.99–1.03)	694	0.99 (0.95–1.03)
Wave 4	1586	**1.04 (1.02**–**1.07)**	1390	1.02 (0.99–1.06)	460	1.05 (0.98–1.13)

Significant estimates in bold

^a^Adjusted for sex and parental education

^b^Adjusted for familial factors by the analyses of discordant twin pairs

Both each one-unit increase in Anxiety/Depressed and Withdrawn/Depressed were associated with DP despite wave of assessment (Table 4). For internalizing behavior problems, the associations were significant for all subscales at each wave except for Somatic complaints at Wave 3. The HRs varied between 1.07 and 1.38 for each one-unit increase on the scales. After adjusting for sex and parental education, the associations remained significant except for Anxiety/Depressed at Wave 1 and Somatic complaints problems at Wave 1 and 3. In the analyses of discordant twins, HRs changed slightly and the significant associations were observed for Withdrawal/Depressed scale at Waves 1–2 and Somatic complaints at Wave 3.

**Table 4:** Crude and adjusted each one-unit increase hazard ratios (HR) and 95% confidence intervals (CI) for disability pension in 2001–2013 following internalizing and externalizing behavior problems during childhood and adolescence

Scale	Crude model for all individuals		Adjusted model for all individuals		Conditional model for discordant twin pairs	
	*n*	HR (95% CI)	*n*	HR^a^ (95% CI)	*n*	HR^b^ (95% CI)
*Internalizing problems*						
Anxiety/depressed						
Wave 1	2074	**1.10 (1.03**–**1.18)**	1792	1.07 (0.97–1.17)	99	1.09 (0.94–1.28)
Wave 2	2155	**1.10 (1.05**–**1.16)**	1876	**1.13 (1.08**–**1.20)**	87	1.13 (0.99–1.29)
Wave 3	2247	**1.08 (1.03**–**1.14)**	1992	**1.10 (1.04**–**1.16)**	88	1.07 (0.98–1.17)
Wave 4	1596	**1.16 (1.10**–**1.22)**	1398	**1.16 (1.08**–**1.24)**	57	1.31 (0.97–1.76)
Withdrawn/depressed						
Wave 1	2078	**1.35 (1.17**–**1.56)**	1796	**1.31 (1.07**–**1.61)**	99	**1.67 (1.09**–**2.56)**
Wave 2	2172	**1.23 (1.10**–**1.38)**	1889	**1.27 (1.11**–**1.45)**	90	**1.35 (1.04**–**1.74)**
Wave 3	2256	**1.20 (1.07**–**1.34)**	1999	**1.27 (1.12**–**1.44)**	89	1.13 (0.92–1.39)
Wave 4	1598	**1.34 (1.19**–**1.52)**	1400	**1.31 (1.12**–**1.53)**	59	1.08 (0.82–1.43)
Somatic complaints						
Wave 1	2083	1.18 (0.99–1.41)	1801	1.06 (0.81–1.39)	99	1.26 (0.78–2.03)
Wave 2	2143	1.10 (0.99–1.23)	1863	1.10 (0.96–1.25)	87	1.24 (0.92–1.68)
Wave 3	2235	1.10 (1.00–1.24)	1982	**1.15 (1.02**–**1.28)**	87	1.25 (0.95–1.64)
Wave 4	1609	**1.19 (1.09**–**1.31)**	1410	**1.21 (1.08**–**1.37)**	59	1.39 (0.96–2.00)
*Externalizing problems*						
Rule-breaking behavior						
Wave 1	2073	1.04 (0.87–1.25)	1791	1.08 (0.85–1.38)	99	0.70 (0.43–1.15)
Wave 2	2153	1.05 (0.94–1.17)	1869	1.04 (0.90–1.20)	89	0.92 (0.71–1.19)
Wave 3	2243	0.98 (0.86–1.12)	1988	0.91 (0.78–1.07)	87	0.94 (0.72–1.22)
Wave 4	1596	**1.17 (1.01**–**1.35)**	1398	1.02 (0.79–1.32)	57	1.18 (0.80–1.72)
Aggressive behavior						
Wave 1	2074	1.01 (0.95–1.06)	1792	1.00 (0.93–1.07)	99	**0.86 (0.75**–**0.99)**
Wave 2	2153	1.02 (0.97–1.08)	1873	1.00 (0.93–1.07)	86	1.06 (0.93–1.22)
Wave 3	2245	0.96 (0.89–1.03)	1991	**0.89 (0.81**–**0.98)**	88	0.96 (0.84–1.10)
Wave 4	1596	**1.13 (1.02**–**1.25)**	1398	1.11 (0.97–1.26)	57	1.21 (0.88–1.67)

Significant estimates in bold

^a^Adjusted for sex and parental education

^b^Adjusted for familial factors by the analyses of discordant twin pairs

We performed additional analyses where behavior problems were entered as dichotomous variables (data not shown). We assigned normalized *T*-scores [21] to differentiate between individuals who had scores in “normal range” (“0”) and who had scores in Clinical/Borderline range of behavior problems, that is, *T*-scores exceeded 65 (“1”). Approximately 8% of the individuals in each wave had behavior problems in Clinical/Borderline range. In the analyses of DP, HRs for the internalizing behavior scale varied between 2.56 and 4.83, whereas for externalizing behavior problems HRs varied between 1.47 and 2.47. In the analyses of SA, HRs were 1.06–1.49 for internalizing behavior problems and 1.32–1.58 for externalizing behavior problems.

## Discussion

The findings of this prospective population-based study were twofold. First, internalizing behavior problems as early as at ages 8–9 years and up to emerging adulthood were associated with future DP. Second, externalizing behavior problems implied an increased risk for SA in young adulthood despite the age of assessment.

Our findings of significant associations between behavior problems during the childhood/adolescence and work disability in early adulthood are partly in line with previous research. Significant associations were found between mental health problems in adolescence and medical benefits (including vocational rehabilitation) received in young adulthood due to reduced work capacity [7]. Another study also reported a higher risk for receipt of medical benefits in young adulthood among those who experienced high levels of anxiety and depression symptoms in adolescence [5]. Our results further demonstrated that the risk for future work disability tends to be elevated after experiencing behavior problems at ages of 8–9 years old. On the other hand, in a follow-up study of young adults that were former patients of child psychiatric clinics, the associations between emotional or conduct disorders and DP in mid-adulthood lacked statistical significance [24]. The discrepancy in findings may be due to the sample characteristics (clinic vs. non-clinic), differences in age span and sample size as well as on how long into adulthood participants were followed.

Internalizing behavior problems in childhood/adolescence were associated with a higher risk for DP, but not with SA in early adulthood. Although CBCL is a screening instrument of behavior problems in a nonclinical sample, some of the participants may have had a more serious psychopathology. We had no knowledge on whether participants were diagnosed with, for example, Autism Spectrum Disorders, a diagnosis that often leads to DP at young ages [25, 26]. Also, internalizing behavior problems in childhood were previously reported to imply higher risk for future mood disorders [10], including depression and anxiety, two of the main DP diagnoses in Sweden as well as in several other Western countries [3]. Interestingly, a previous study reported a significant association between lifetime internalizing disorders (measured in adulthood) and sick leave due to mental diagnoses [27]. In the present study, the associations were estimated for SA due to any diagnosis which may contribute to differences in findings.

Rule-breaking behavior, a subscale of externalizing behavior problems, was shown to be associated with future SA. This finding may sound unexpected as rule-breaking behavior can hardly be a diagnosis behind SA. However, this association becomes rather anticipated in the light of the previous studies. First, externalizing and internalizing behavior problems have repeatedly been shown to be comorbid and partly share the same etiology [28]. Thus, possible diagnoses behind SA among people with high levels of externalizing behavior problems may include those related to internalizing problems, including depression or anxiety. Second, externalizing behavior problems in adolescence have previously been shown to be a risk factor for a wide range of mental disorders in adulthood, including mood and disruptive disorders, as well as physical health outcomes [10, 11, 29]. Previous research has also highlighted the different etiology of aggressive and rule-breaking behavior, also referred as to physically aggressive (e.g., fighting, bullying) and non-aggressive rule-breaking behavior (e.g., stealing, lying), respectively [30]. Those with aggressive behavior problems are usually early starters and were linked to antisocial personality disorder in adulthood, whereas rule-breaking starts usually in adolescence and was linked to higher risk for substance abuse [31]. A few studies have reported that adolescent rule-breaking, but not aggressive, behavior tend to increase the risk for mental health problems in adulthood [10, 29], whereas another study showed significant association between adolescent aggressive behavior (or conduct disorder) and future psychopathology [11]. Our findings suggest that rule-breaking, and not aggressive, behavior tends to increase risk for SA in adulthood.

The results showed a tendency that internalizing behavior problems were associated to DP, whereas externalizing behavior problems were associated to SA. These findings suggest that there might be different pathways leading to SA and DP. Both SA and DP are related to work incapacity, temporary or permanent, due to disease or injury. A process leading to DP is usually several-years long and is often preceded by long-term SA. Thus, one could expect that pathways leading to SA and DP could partly overlap. However, in Sweden, young adults up to age 29 years who are diagnosed with severe diagnoses can be granted DP without having any preceding SA spell. Thus, our findings of the association between internalizing behavior problems and DP may be influenced by the severity of diagnosis which we had no possibility to adjust for. Future studies are needed to shed more light on the underlying mechanisms of the studied associations.

The results of the present study suggest that behavior problems experienced during adolescence may increase risk for work incapacity in adulthood. Before the results are replicated in future studies, we can only speculate on how our findings could be interpreted in terms of prevention and intervention of work incapacity. On one hand, paying efforts to prevent or reduce behavior problems at early ages could help to reduce work incapacity in adulthood. On the other hand, the intervention strategies could also target the consequences of the behavior problems and include, for example, adjustment of working conditions to young adults experiencing behavior problems.

The analyses of discordant twins showed slightly attenuated estimates, suggesting that familial factors played a minor role for the studied associations. That is, factors that are unique to each individual and not shared with a co-twin seem to primarily influence the reported associations. However, the results should be interpreted with caution due to the low number of discordant twin pairs, especially in the analyses of DP, and the relatively low HRs.

The estimates of significant associations between internalizing behavior problems and DP were approximately of the same size irrespective of the age of assessment. Bearing in mind that the follow-up time started directly after the assessment of behavior problems at Waves 3 and 4, one would perhaps expect that the HRs should be higher the closer the assessed behavior problems was to adulthood. However, our findings are consistent with previous studies reporting moderate stability of behavior problems during childhood and adolescence, e.g., [32].

The estimates of the associations may seem consistently small and the clinical significance of our results may be questioned. However, the estimates imply an increase in hazard following each one-unit increase in the behavior problems. Since the total scores of CBCL scales varied between 10 and 22 scores, even a low HR would suggest a noteworthy risk for those individuals with high scores.

### Strengths and limitations

The study has several strengths, including the longitudinal population-based design and nationwide register data with no loss to follow-up. The individuals were followed for up to 20 years since the first assessment of behavior problems was conducted when the twins were 8–9 years old. This gave us a unique opportunity to investigate long-term effects of behavior problems in childhood and adolescence on both SA and DP. Some limitations should also be addressed. First, the number of DP cases observed during the follow up was low. Thus, the results should be interpreted with caution and need to be replicated using larger samples. Second, due to the few people that were granted DP, the analyses were adjusted for, instead of stratified by sex. Previous research has consistently shown sex differences in frequency of occurrence of internalizing and externalizing behavior problems in adolescence (e.g., [33], as well as in being grated DP [34]. Thus, it is possible that the associations in the present study would be different and/or significant if estimated separately among women and men. Third, only parent-reports of behavior problems were available at Wave 1 and were used in the analyses, whereas self-reported data were used at Waves 2–4. Agreement between parental and child ratings for CBCL symptoms was previously reported to be rather low [35]. Thus, differences in the estimates between those at Wave 1 and those at later Waves might be due to different reporters rather than due to changes in the levels of problem behavior at different ages. Fourth, only SA spells longer than 14 days could be included, which can be seen both as strength and a limitation. Fifth, SA benefits can be granted only the individuals having income from work or unemployment benefits. In the present study, the participants were followed from the years they turned 16 and up to 28–29 years old. Approximately 40% of all individuals born in Sweden 1985–1986 began their higher education studies when they were 24 years old at the latest (Higher Education in Sweden, 2016). Thus, a selection bias may be present in our study as participants that were students and did not work during the follow-up were not at risk for SA. However, at ages of 25–26 years, 75% of the respondents were registered as having income from work (>10,700 Swedish crowns/year) and hence eligible for sickness absence benefits. Sixth, the estimates are shown for one-point change in behavior scores. When comparing between different ages, the one-point change in behavior score at one wave may be different from the change at another wave due to different variability at different waves. However, the variability for externalizing scores show only minor changes between the waves and thus the one-point change in behavior scores is reasonable to compare between the waves. As only twins born in Sweden were included, the results might not be generalizable to immigrants. Lastly, the response rate reached only 52% at Wave 4 and, therefore, the significance of associations could be affected by that.

## Conclusions

Disability pension in young adulthood was predicted by internalizing behavior problems in childhood and adolescence, whereas externalizing behavior problems were associated with sickness absence. If confirmed in future studies, the results suggest that early prevention and intervention efforts to reduce behavior problems may promote a successful start in working life.
